# Editorial: Immune Regulation in Kidney Diseases: Importance, Mechanism and Translation

**DOI:** 10.3389/fmed.2021.616880

**Published:** 2021-02-11

**Authors:** Cheng Yang, Songjie Cai

**Affiliations:** ^1^Department of Urology, Zhongshan Hospital, Fudan University, Shanghai, China; ^2^Shanghai Key Laboratory of Organ Transplantation, Shanghai, China; ^3^Zhangjiang Institute of Fudan University, Shanghai, China; ^4^Transplantation Research Center, Renal Division, Brigham and Women's Hospital and Harvard Medical School, Boston, MA, United States

**Keywords:** kidney, immune regulation, acute kidney injury, ischemia reperfusion injury, transplantation—kidney, chronic kidney disease, erythropoietin

Immune system is the vital system in humans. Our defense to infection relies on the system, which is regulated precisely. In our immune system, it exists a balance between offensive and defensive powers. The attack by immune cells cannot be amplified infinitely. Uncontrollable immune response will elicit autoimmune disease. On the other hand, weak immune response cannot kill tumor cells. Therefore, immune regulation by many regulatory immune cells and cytokines is of importance in physiological and pathological condition. The kidneys, both native and transplant, are closely related with the immune system. In this Research Topic, we bring you some excellent original studies, reviews, and case reports.

In this issue, these are three case reports related on kidney transplantation. In kidney transplantation, ischemia reperfusion (IR) injury is an evitable process, which is associated with delayed graft function, rejection, and long-term allograft survival. The immune system participates in the IR injury in terms of innate immunity activation, inflammation and stress. In previous study, He et al. established a novel procedure called ischemia-free liver transplantation during which the donor livers can be procured, preserved, and implanted without cessation of oxygenated blood supply to the grafts. In this issue, their team reported the first case of ischemia-free kidney transplantation (IFKT) in human. In brief, the left kidney was procured after ligating the left renal artery and vein and immediately cold flushed through the left kidney artery. This graft was preserved in ice-cold University of Wisconsin solution. The right kidney was subjected to IFKT. This method provides a unique solution to reduce IR injury or delayed graft function morbidity (He et al.). The second case by Zhang G. et al. reported a patient with combined liver-pancreas-kidney transplantation who had been followed up by 14 years. This patient suffered from end-stage liver disease, post-chronic hepatitis B, cirrhosis, chronic renal failure, and insulin-dependent diabetes mellitus caused by chronic pancreatitis. In 14 years of follow-up, no severe rejection or other complications were observed. Simultaneous piggyback orthotopic liver and heterotopic pancreas-duodenum and renal transplantation is a good therapeutic option for patients with exocrine pancreatic insufficiency and insulin-dependent diabetes combined with hepatic and renal failure (Zhang G. et al.). In kidney transplant patients, immunosuppression therapy is inevitable. The immune system is generally suppressed because all immunosuppression drugs are not specific to the allografts. Therefore, infection after transplantation is a very common but complicated problem. Opportunistic infections such as fungal is not rare in kidney transplant patients. In this case, Fumet et al. reported a patient with *pulmonary mucormycosis* 38 days after kidney transplantation (1). This case is a reminder that early diagnosis of fungal infection is imperative.

Acute kidney injury (AKI) brings heavy burden to the healthcare system with high morbidity and mortality. AKI occurs in about 13.3 million people per year, 85% of whom live in the developing world, and, although no direct link between AKI and death has yet been shown, AKI is thought to contribute to about 1.7 million deaths every year (2). Therefore, it is urgent to discover novel useful biomarkers and drugs of AKI. N-terminal pro-B-type natriuretic peptide (NT-proBNP) is a useful cardiac biomarker that is associated with acute kidney injury (AKI) and mortality after cardiac surgery. However, its prognostic value in cardiac surgical patients receiving renal replacement therapy (RRT) remains unclear. In this study, Su et al. established a prediction model based on NT-proBNP in cardiac surgical patients receiving RRT. The area under the receiver operating characteristic curve of NT-proBNP before surgery, at RRT initiation, and on the first day after RRT for predicting 28-day mortality was 0.64, 0.71, and 0.68, respectively (Su et al.). This study demonstrated that serum NT-proBNP was an independent predictor of 28-day mortality in cardiac surgical patients with AKI requiring RRT.

As mentioned above, no specific and effective therapy is available for AKI. In this issue, researchers investigated several novel molecule and peptide drugs, as well as cell therapy. Previous study found that 3-Deazaneplanocin A (DZNep), an inhibitor of Ezh2, had an inhibitory effect on graft-vs.-host disease (GVHD) in a kidney or bone marrow transplantation model, and it can only induce apoptosis of activated T cells but has no effect on naïve T cells. In this study, Li J. et al. further investigated the role of DZNep in kidney IR injury. They demonstrated that DZNep alleviated renal IR injury in mice by inhibiting T cell activation through direct and indirect pathways. The indirect pathway may involve the impairment of interactions between T cells and macrophages by the TIM-1–TIM-4 axis, suggesting that DZNep can be a novel strategy for preventing renal IRI following kidney transplantation (Li J. et al.). Erythropoietin (EPO), an evolutionarily conserved hormone mainly produced in the kidney, has been well-documented for its indispensable role in erythropoiesis. In recent years, numerous studies have shown that EPO acts far beyond erythropoiesis (3). EPO modulates immune cells function and protects tissue against injury. Our group synthesized a series of cyclic analogs and found that the head-to-tail thioether-cyclized HBSP, namely, cyclic helix B peptide (CHBP), remained stable in human plasma and had a 2.5-folds longer half-life than its counterpart in human hepatocytes (4). Zhang Y. et al. demonstrated that CHBP reduced endoplasmic reticulum stress through CHOP/PERK/JNK pathway. CHBP also inhibited the increase of CHOP protein, not only in tubular epithelia cells, but also in the IR injury kidneys at 2 weeks. Moreover, CHBP reduced the expression of PERK mRNA and protein, JNK and HMGB-1 protein, as well as early and later apoptosis. This study revealed a novel mechanism of CHBP in AKI, and CHOP might be a potential biomarker of IR-induced AKI (Zhang Y. et al.).

Mesenchymal stem cells (MSCs) have been found to exert several biological functions, such as repairing tissue damage, suppressing inflammatory responses, and modulating the immune system. Exosomes originated from mesenchymal stem cell were reported to activate signaling pathways by binding to receptors. In this issue, Li L. et al. found that exosomes from MSCs ameliorated kidney tubular epithelia cell apoptosis and interstitial inflammation through NK-κB pathway. Although the efficacy of molecular hydrogen (H_2_) in IR injury has been reported, oral intake of H_2_-rich water and inhalation of H_2_ gas are still not widely used in clinical settings because of the lack of efficiency and difficulty in handling. Kawamura et al. successfully generated large quantities of H_2_ molecules by crushing silicon (Si) to nano-sized Si particles (nano-Si) which were allowed to react with water. They investigated whether oral administration of H_2_ could ameliorate kidney IR injury. In a rat kidney IR model, oral nano-Si intake downregulated immune response, cytokine production, and extrinsic apoptosis. This study might provide a novel gas therapy for renal IR injury (Kawamura et al.). As we know, hepatorenal syndrome is a complicated disease in clinic. The liver function is often compromised in patients with AKI and in animal models. However, the underlying mechanisms are not fully understood. Here, Shang et al. found that renal IR injury elicited acute liver injury and inflammation response. They demonstrated that renal IR can directly activate NF-kB and induce acute production of proinflammatory cytokines in the liver. Renal IR-induced hepatic inflammatory response may contribute to impaired liver function and systemic inflammation (Shang et al.).

Besides AKI, there are two researchers about chronic kidney disease (CKD). Chronic low-grade inflammation is a major stimulus for progression of CKD in individuals consuming high-fat diet. Lingonberry is rich in anthocyanins with demonstrated anti-inflammatory effect. Madduma Hewage et al. investigated the potential renal protective effect of lingonberry and its anthocyanin (cyanidin-3-glucoside) in high-fat diet fed obese mice and in human proximal tubular cells. They found that lingonberry supplementation can reduce inflammatory response, suggesting lingonberry might be a complementary fruit for chronic kidney disease (CKD) therapy (Madduma Hewage et al.). Since chronic inflammation and immune system dysfunction play a key role in the pathogenesis of CKD, Xiang et al. explored the genome-wide expression profile in human peripheral blood T cells under stimulation by indoxyl sulfate (IS) which was one of the protein-bound renal toxins. A total of 5,129 DEGs were identified, and IS may influence multiple biological functions of T cells including inflammatory response and cell cycle regulation. This study provided an immune regulation network in T cells stimulated by IS (Xiang et al.). IgA nephropathy is a common factor of end stage renal disease in China. A variety of immune cells (e.g., dendritic cells, NK cells, macrophages, T-lymphocyte subsets, and B-lymphocytes, etc.) and molecules (e.g., IgA receptors, Toll-like receptors, complements, etc.) in innate and adaptive immunity are involved in the pathogenesis of IgA nephropathy. Chang and Li reviewed the role of immune regulation in IgA nephropathy. This review focus on both innate and adaptive immunity, as well as mucosal immunity (Chang and Li).

In the field of kidney transplantation, there are two original researches. Liao et al. retrospectively enrolled fifty-six patients with biopsy-proved rejection or non-rejection and 11 stable allograft function patients. Both soluble CD146 in plasma and local CD146 expression in kidney allografts were detected. They found plasma soluble CD146 could be a biomarker of acute rejection after kidney transplantation, whose area under the receiver operating characteristic curve was 0.895 (Liao et al.).

In conclusion, all published articles covered immune regulation in acute, chronic kidney injury and kidney transplantation. We believe this Research Topic can provide new findings of immune regulation in kidney diseases. Thanks to all contribution authors, reviewers and editors.

## Author Contributions

CY and SC wrote the editorial. Both authors contributed to the article and approved the submitted version.

## Conflict of Interest

The authors declare that the research was conducted in the absence of any commercial or financial relationships that could be construed as a potential conflict of interest.
